# The Effects of Various Mouthwashes on Osteoblast Precursor Cells

**DOI:** 10.1515/biol-2019-0042

**Published:** 2019-07-22

**Authors:** In-Seok Song, Ji Eun Lee, Jun-Beom Park

**Affiliations:** 1 Department of Periodontics, Seoul St Mary's Hospital, College of Medicine, The Catholic University of Korea, 222, Banpo-daero, Seocho-gu, Seoul, 06591, Republic of Korea; 2 Department of Oral and Maxillofacial Surgery, Korea University Anam Hospital, Seoul, 02841, Republic of Korea; 3 Department of Periodontics, College of Medicine, The Catholic University of Korea, Seoul, 06591, Republic of Korea

**Keywords:** mouthwashes, osteoblasts, cell survival

## Abstract

This study examined whether or not various mouthwashes have significant effects on the viability or morphology of mouse osteoblast-like cells. Mouse calvarial preosteoblast cells were cultured and prepared, then treated with a 0.12% chlorhexidine digluconate solution containing essential oils with or without alcohol, and a cetylpyridinium chloride solution, and sodium fluoride, respectively. Each well was treated with one of six mouthwashes for either 30 sec, 1.5 min, or 4.5 min. The viability of the treated cells was quantitatively analyzed by a Cell Counting Kit-8. The viability of osteogenic progenitor cells decreased significantly irrespectively of the types of mouthwashes. The changes of cell morphology were seen in all groups of mouthwashes; however, they were more noticeable on the chlorhexidine digluconate-treated group. A progressive increase in treatment time over 30 sec did not seem to deteriorate cellular viability. There was no significant difference in viability or morphological change between different formulations of the same brand. Although various mouthwashes without alcohol as an ingredient are available, nonalcoholic mouthwashes were not likely to be less harmful to the cells. Collectively, commercially available mouthwashes could inhibit cell viability and alter the morphology of osteoblastic precursor cells irrespectively of brands, treatment time, or alcohol content.

## 1 Introduction

Various mouthwashes are commercially available over the counter. Chlorhexidine digluconate (CHX) is the most commonly prescribed antibacterial gargling agent, a bisbiguanide that inhibits and prevents bacteria forming by binding to cell membranes and increasing permeability and leakage of intracellular components. Its effect is mainly due to substantivity in the mouth [1]. Listerine^®^ (LIS) is phenolic essential oils combined with thymol, eucalyptol, menthol, and methylsalicylate in an alcohol vehicle. The mechanism is through protein denaturation of the bacterial membrane and inhibition of enzyme activity. LIS is a strong antimicrobial mouthwash and is frequently used in many dental fields such as orthodontic bracket disinfection [2]. Garglin^®^ (GGN) is a popular mouthwash used for decades in the Republic of Korea. GGN consists of cetylpyridinium chloride (CPC) as an active ingredient, a cationic quaternary ammonium compound that is cytotoxic to bacterial and other microorganisms. It has been shown to be effective in preventing plaque accumulation and decreasing gingivitis [3, 4, 5].

Although these mouthwashes are effective antiseptics, there is a concern about an adverse effect on various mammalian cells. For example, previous studies have demonstrated the cytotoxicity of CHX on oral mucosal fibroblast may be increased in the concentration and time-dependent manner [6]. Clinical use of 0.12% CHX twice daily over eight days caused DNA damage on oral mucosal cells *in vitro* [7]. An inhibitory effect of mouthwashes has been shown in osteoblasts and osteoclasts [8,9]; however, there is a lack of thorough information on whether or not these agents can harm bone formation and hinder periodontal bone regeneration. Therefore, this study investigated the effects of the aforementioned mouthwashes on osteoblast-like cells by examining cell morphology and viability.

## 2 Materials and methods

### 2.1 Cell culture

Osteoblast-like cells (mouse calvarial preosteoblast cells, MC3T3-E1, American Type Culture Collection, Manassas, VA, USA) were plated at 96-well at a density of 6.25 x 10^3^ cells/well and maintained in α-minimum essential medium (αMEM, Welgene, Daegu, Korea) supplemented with 10% fetal bovine serum (Thermo Scientific, Logan, UT), antibiotics (penicillin 100U/mL and streptomycin 100 μg/mL (Gibco, Invitrogen, Carlsbad, CA)). The cultures were kept in a humidified atmosphere with 5% CO_2_ and 95% air at 37°C for 24 hours.

### 2.2 Preparation with mouth rinse and evaluation of cellular morphology

**Figure 1:**
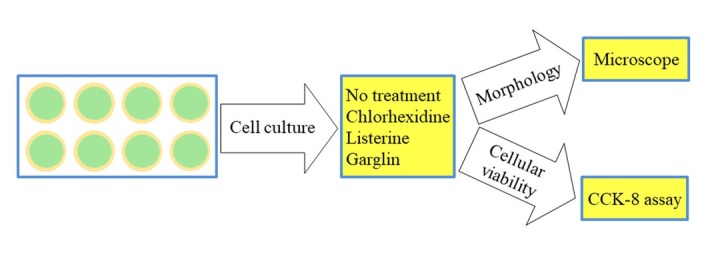
Diagram showing the overview of the study design.

Figure 1 shows the overview of the study design. Six mouthwashes were applied for this study: (1) a 0.12% chlorhexidine digluconate solution (CHX, Hexamedine, Bukwang, Seoul, Korea); (2) a solution containing essential oils (LIS Citrus, Listerine^®^ Citrus, Johnson & Johnson, Bangkok, Thailand); (3) a solution containing essential oils without alcohol (LIS Zero, Listerine^®^ Zero, Johnson & Johnson); (4) Garglin^®^ Regular containing CPC (GGN, Dong-A Pharmaceutical Co., Seoul, Korea); (5) Garglin^®^ Medical containing essential oils (GGN Med, Dong-A Pharmaceutical Co.); and (6) Garglin^®^ Child containing sodium fluoride (GGN Child, Dong-A Pharmaceutical Co.). Each well was treated with one of the six mouthwashes for 30 sec, 1 min and 30 sec (1.5 min), or 4 min and 30 sec (4.5 min). An untreated culture well served as a control. The morphological changes were observed under an inverted microscope (Leica DM IRM, Leica Microsystems, Wetzlar, Germany) after each treatment.

### 2.3 Quantitative determination of cellular viability

The viability of the treated cells was quantitatively analyzed by a Cell Counting Kit-8 (Dojindo molecular technologies Inc., Rockville, MD). A water-soluble tetrazolium salt-8 (2-(2-methoxy-4-nitrophenyl)-3-(4-nitrophenyl)-5-(2,4-disulfophenyl)-2H-tetrazoium, monosodium salt) solution was added and incubated for 12 h. The amount of generated formazan was determined by reading the absorbance at a 450 nm wavelength using the microplate spectrophotometer system (BioTek, Winooski, VT).

### 2.4 Statistical analysis

The data are represented as the means ± standard error of the mean of the experiments. A test of normality and the equality of variances in the samples was conducted. A two-way analysis of variance (ANOVA) was used for the evaluation of the effects of application time and types of gargles. A one-way ANOVA with Tukey’s post hoc test was performed to determine the differences between the application time in each group using a commercially available program (SPSS 12 for windows, SPSS Inc., Chicago, IL, USA) with the level of significance at 0.05.

## 3 Results

### 3.1 Evaluation of cellular morphology

**Figure 2:**
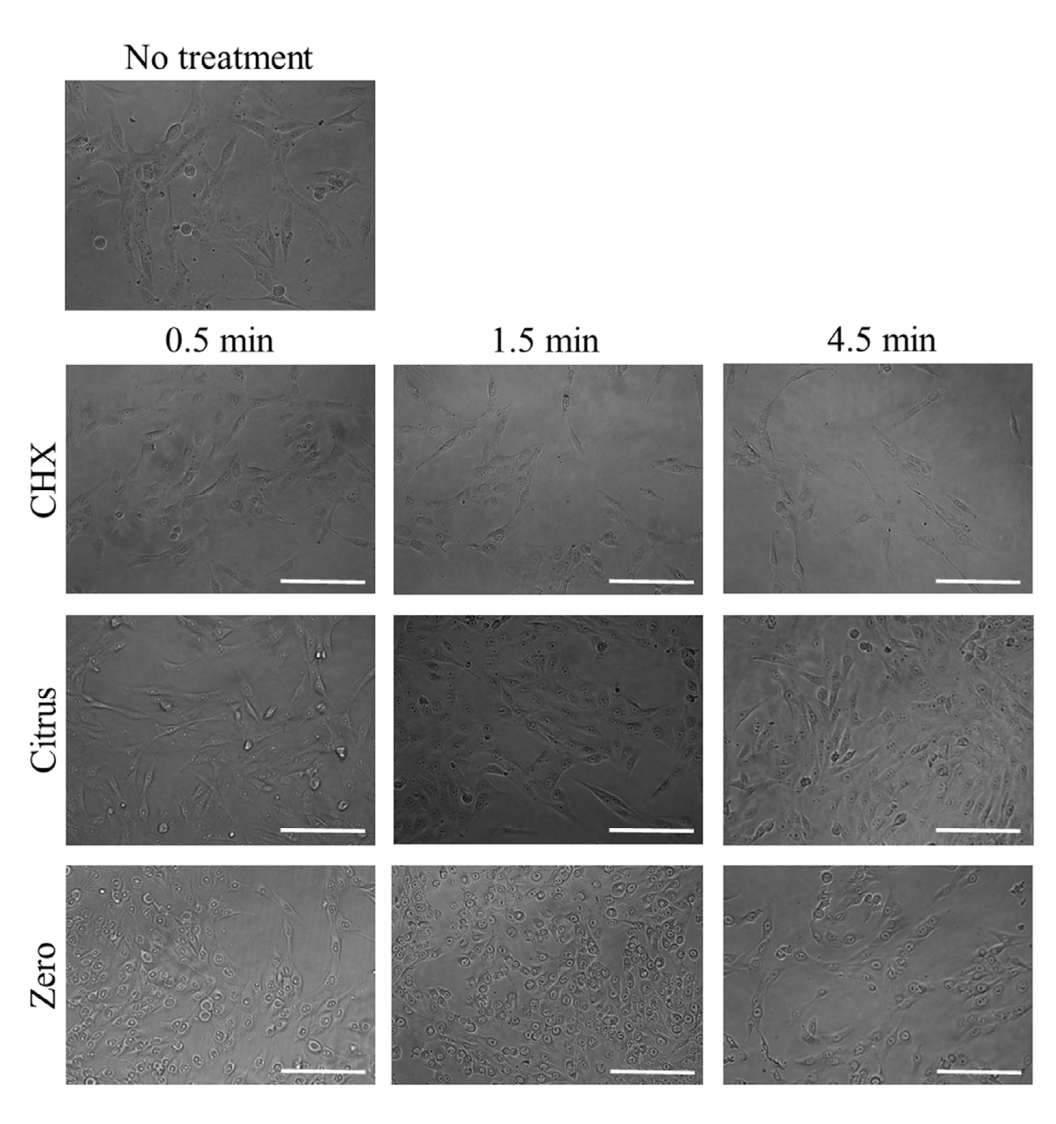
Evaluation of cellular morphology after treatment with 0.12% CHX, LIS Citrus, and LIS Zero. The scale bar indicates 200 μm.

In the microscopic evaluation, the untreated cells attached to the culture plate showed a well-organized actin cytoskeleton. The treatment of the adult stem cells with 0.12% CHX produced a more rounded shape (Figure 2). Treatment for a longer time caused a more noticeable alteration, with 0.12% CHX. Similar trends were achieved in the LIS Citrus and LIS Zero groups. We noticed alteration in the cytoskeletal organization and observed the rounding up of the cells or progressive detachment from the substrate for the experiments.

### 3.2 Cellular viability

**Figure 3:**
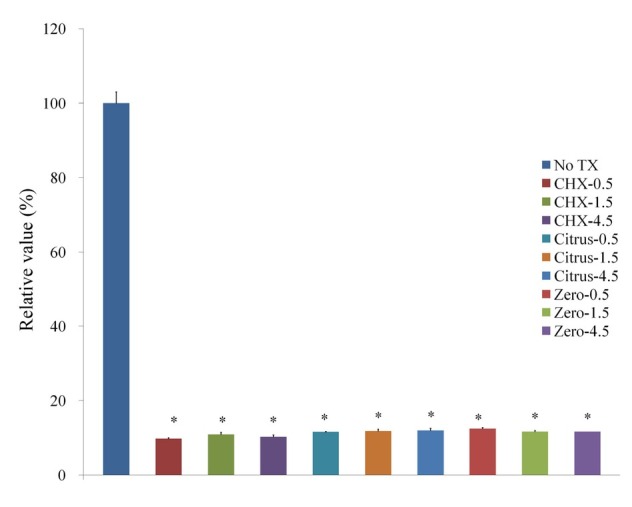
Cellular viability using Cell Counting Kit-8 after treatment with 0.12% CHX, LIS Citrus, and LIS Zero.

The Cell Counting Kit-8 assay showed that the treatment with CHX, LIS Citrus, and LIS Zero groups affected cell viability (Figure 3). The CHX, LIS Citrus, and LIS Zero showed cytotoxic effects on osteoblast-like cells *in vitro*, with a mean viability of 9.8% ± 0.2%, 11.6% ± 0.1%, and 12.5% ± 0.3%, respectively, after exposure for 30 sec when the control group was considered 100% (100.0% ± 3.0%). The progressive increase in the treatment time of CHX, LIS Citrus, and LIS Zero up to 4.5 min did not induce significant decreases of viability. The mean cell viability for CHX was 10.9% ± 0.5% and 10.3% ± 0.9% for 1.5 and 4.5 min (*P=*0.05). The viability of LIS Citrus for 1.5 min and 4.5 min was 11.8% ± 0.5% and 11.9% ± 0.6%, respectively (*P=*0.05). The mean cell viability for LIS Zero was 11.6% ± 0.3% and 11.6% ± 0.0% for 1.5 and 4.5 min (*P=*0.05).

**Figure 4:**
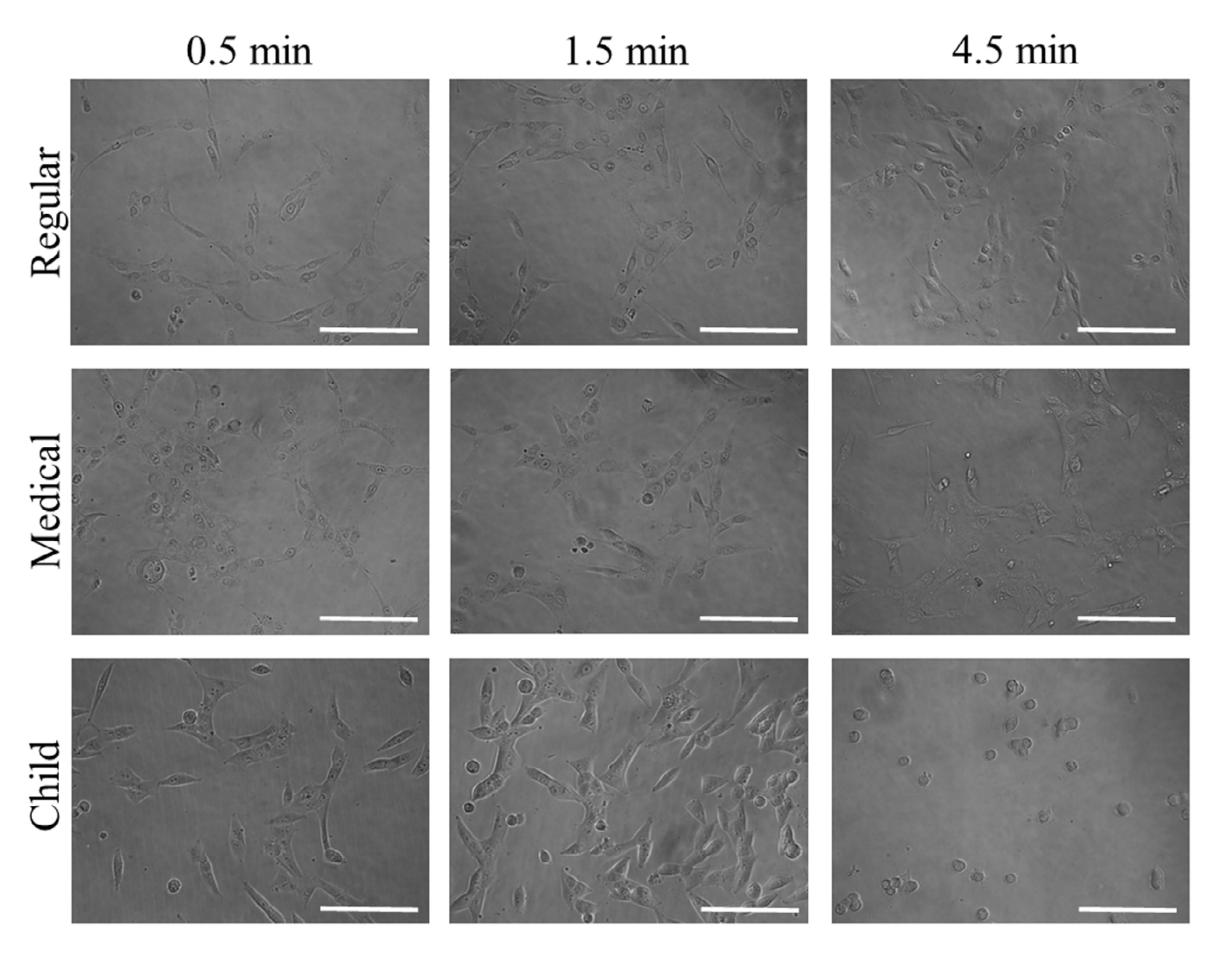
Evaluation of cellular morphology after treatment with Gargin^®^ formulations (GGN, GGN Med, and GGN Child).

**Figure 5:**
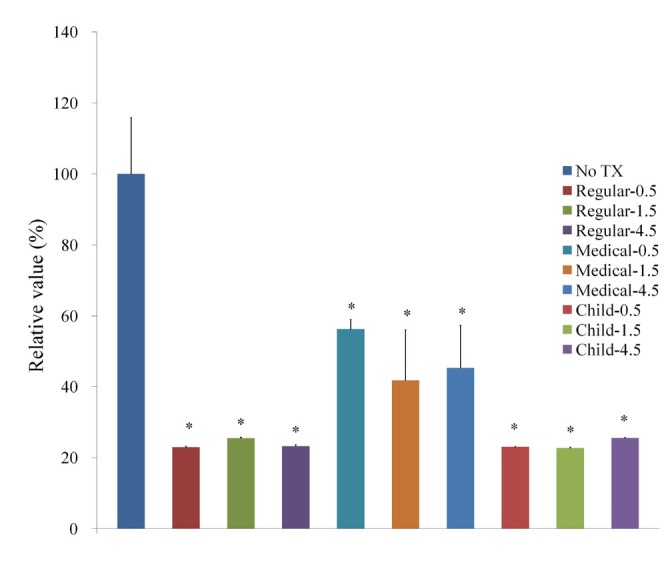
Cellular viability using Cell Counting Kit-8 after treatment with Gargin^®^ formulations.

Cellular morphology after treatment with GGN formulations (Garglin^®^ Regular, Garglin^®^ Medical, and Garglin^®^ Child) is shown in Figure 4. Alteration in the cytoskeletal organization was noted irrespective of GGN formulations. The treatment with GGN formulations affected cell viability (Figure 5).

## 4 Discussion

This study showed that all of the mouthwashes—CHX, LIS, and GGN—inhibited viability and deformed the morphology of mouse calvarial preosteoblast cells. The progressive increase in the treatment time of CHX, LIS, and GGN up to 4.5 min did not induce significant decreases of viability compared to the 30 sec group. Alcohol adjuvant was not likely to affect the cytotoxicity of the mouthwashes, irrespective of brand.

A comparison between mouthwashes was performed previously. A study showed that CHX reduced the proliferation of gingival fibroblast in a dose-dependent manner [1]. The 0.12% CHX totally inhibits cell proliferation, and even 0.01% CHX inhibits cell proliferation by 50%. The dilution of essential oil (LIS), however, did not result in an antibacterial effect as much as with CHX. Also, Listerine® did not show sustained toxic effects on fibroblasts as much as CHX. Another comparative study showed that 0.2% CHX was more cytotoxic to LIS cool mint on human gingival fibroblasts [10]. Similarly, the antibacterial effect of CHX is the most powerful, followed by LIS in terms of viability of plaque bacteria [11]. A comparison of the antiseptic effects between LIS and CPC was performed previously. There was no statistically significant difference in prevention of the plaque and gingivitis benefits between the CPC mouth rinse and the EO mouthwash [3]. Another study demonstrated the superiority of daily use of LIS compared to 0.05% CPC mouthwash in decreasing plaque and gingivitis [12].

Because the cytotoxicity of mouthwashes is indiscriminate, we investigated adverse effects on various cells. Inhibition of oral fibroblastic wound healing [7,13, 14, 15] and odontoblastic tooth repair [16, 17] by CHX were noted as well as an antiseptic effect on microorganisms [18,19]. Another study reported toxic effects of CHX on human periodontal ligament cells [20]. Deletion of glutathione, which has a role in cellular protection from damage from reactive oxygen species, was proposed to be the mechanism of the CHX-induced cytotoxicity [21].

As for toxic effects on bones, CHX can have propound cytotoxic activity to osteoblast-like cells. CHX reduced the cell viability and differentiation potential *in vitro* on human osteoblastic cells from periodontium [22,23]. Similarly, the application of 0.12% CHX during a 2-min period immediately led to human alveolar bone cell destruction [19]. Another report for the human osteosarcoma cells revealed that CHX induced mitochondrial dysfunction, an increase in intracellular calcium ion, and oxidative stress, which resulted in apoptotic and necrotic cell deaths [24].

Thymol, one of the active ingredients of the essential oils (LIS), induced numerical chromosome abnormalities in rat bone marrow cells in a dose-dependent manner and inhibited the mitotic index irrespectively of treatment time or concentration of thymol [25]. Other studies about various cell lines, including murine melanomas [26] and human colon adenocarcinoma cells [27], showed significantly reduced cell viability or induced an antitumor effect by thymol. This study also found a cytotoxic effect of LIS Citrus and LIS Zero after treatment for 30 sec or more. Therefore, we could postulate that LIS, through active ingredients including thymol, can be a potent inhibitor for bone regeneration.

Previous clinical studies showed positive antiplaque activity and reduction of gingivitis/gingival bleeding with regular use of CPC, an ingredient of GGN, after tooth brushing for 6-month clinical trials [28,29]. A systematic review demonstrated that CPC mouth rinse, when used for adjuncts, provides a small but significant beneficial effect in reducing plaque and gingivitis [5]. The CPC is more cytotoxic to oral fungus than CHX [30]. Systematic reviews revealed that CPC may also be effective in reducing oral malodor [31, 32, 33]. However, there are few previous studies of CPC regarding its effect on osteoblast. Interestingly, one study showed CPC inhibition of osteoclast differentiation by suppressing the RANKL-induced expression of c-Fos and NFATc1 via ERK and NF-*κ*B pathways [9]. They suggest that by modulation of RANKL/RANK signaling, and subsequent osteoclast inhibition, prevention of periodontal bone resorption would be possible.

The results of this study suggest that careful use of mouthwash is necessary when a planned intra-wound application such as guided bone or tissue regeneration because the aforementioned mouthwashes can harm the bone formation. Harvested bone soaked and cleaned with CHX showed total cell death, supporting this finding [34]. Interestingly, CHX can be applied for periodontal regeneration if it is used at very low concentrations or slow-release kinetics [19,35]. Unless the mouthwashes are diluted at a very low concentration, they should be used with caution on the bone.

This study demonstrated no significant difference of cytotoxicity between the 30-s, 1.5-min, and 4.5-min groups, irrespective of mouthwashes. Similar experiments were performed for the stem cells from buccal fat pads, which showed reduced cell viability after CHX or LIS treatment for either 30 s, 1.5 min, or 4.5 min, but no significant decrease in viability from 30 sec up to 4.5 min [36]. These results were contradictory to previous studies. CHX was cytotoxic to human periodontal ligament (PDL) cells [20], odontoblast-like cells [16], and stem cells from human exfoliated deciduous teeth in dose and time-dependent manners [37]. A report showed that although 0.12% (1.2 mg/ml) CHX has a strong cytotoxicity and DNA cell damage compared to other agents due to substantivity, this effect is not harmful to epithelial wound healing because CHX does not penetrate the basal layer of the epithelium [6]. Collectively, the responses against mouthwashes seem to differ depending on the type of cells.

Ethanol in mouthwashes can be used as a preservative, stabilizer, solubilizer, sensory cue with a distinctive taste, and antiplaque efficacy enhancer (adjuvant effect). Ethanol and methylsalicylate components in the LIS deliver strong antioxidant traits [38]. LIS can act as an antioxidant to eradicate the negative effects of oxygen-free radicals on epithelial cells and maintain epithelialization of the wound. Some formulas without alcohol have recently become available because of bad taste, mucosal irritation, fear of cancer, etc. There has been concern about whether use of mouthwashes containing alcohol increases the prevalence of oral cancer [39] because alcohol consumption is a known risk indicator for oral cancer [40]. However, a recent systematic review demonstrated that there is no association between the use of mouthwashes containing alcohol and oral cancer [41]. This study also failed to find differences of cytotoxicity to osteoblast-like cells between alcohol and nonalcoholic mouthwashes within the same brand.

This study has some limitations; First, it is confined to a laboratory environment that may be different from the real oral environment. Further, *in vivo*, or human clinical trials, would be required to elucidate the efficacy and safety of mouthwashes. Second, various stem or progenitor cells can involve periodontal regeneration, therefore it would be necessary to examine other precursor cells. This study also has beneficial points; We compared several types and subtypes of mouthwashes, suggesting evidence of safety, and offering guidelines for adequate treatment time.

In conclusion, commercially available mouthwashes could inhibit cell viability and alter morphology of osteoblastic precursor cells irrespectively of brand, treatment time, or alcohol content.
